# Supplementary material for: Second quantization of many-body dispersion interactions for chemical and biological systems
Source: Nat Commun. 2023 Dec 12;14:8218. doi: 10.1038/s41467-023-43785-z (PMC10716193; doi:10.1038/s41467-023-43785-z)
Supplement: Supplementary file 1 — Supplementary Information [file 41467_2023_43785_MOESM1_ESM.pdf]

Supplementary Discussion for " Second Quantization of Many-Body Dispersion Interactions for Chemical and Biological Systems"

Matteo Gori,[1,2] Philip Kurian,[2] and Alexandre Tkatchenko[1]

[1]*Department of Physics and Materials Science, University of Luxembourg, L-1511 Luxembourg City, Luxembourg*

[2]*Quantum Biology Laboratory, Howard University, Washington DC 20060, USA*

I. BOGOLIUBOV TRANSFORMATION FOR QUANTUM DRUDE OSCILLATORS (QDOS) IN THE SQ-MBD FRAMEWORK

It can be easily checked that such $3N \times 3N$ real matrices X, Y satisfy the Bogoliubov conditions assuring preservation of the canonical commutation relations for the sets of creation/annihilation operators [S1],

$$\begin{aligned} XX^\dagger - YY^\dagger &= \mathbb{I}_{3N\times 3N}, \qquad YX^{\mathrm{T}} - XY^{\mathrm{T}} = 0 \\ X^*X^{\mathrm{T}} - Y^*Y^{\mathrm{T}} &= \mathbb{I}_{3N\times 3N}, \qquad Y^*X^\dagger - X^*Y^\dagger = 0\ . \end{aligned} \tag{S1}$$

The conditions in the first and second lines of Eq. (S1) are equivalent in the case of real matrices. In fact,

$$\begin{aligned} &XX^{\mathrm{T}} - YY^{\mathrm{T}} = \frac{1}{4}\Big[(\tilde{\mathcal{D}}^{1/2}\mathcal{O}\mathcal{D}^{-1/2} + \tilde{\mathcal{D}}^{-1/2}\mathcal{O}\mathcal{D}^{1/2})(\mathcal{D}^{-1/2}\mathcal{O}^{\mathrm{T}}\tilde{\mathcal{D}}^{1/2} + \mathcal{D}^{1/2}\mathcal{O}^{\mathrm{T}}\tilde{\mathcal{D}}^{-1/2}) + \\ &- (\tilde{\mathcal{D}}^{1/2}\mathcal{O}\mathcal{D}^{-1/2} - \tilde{\mathcal{D}}^{-1/2}\mathcal{O}\mathcal{D}^{1/2})(\mathcal{D}^{-1/2}\mathcal{O}^{\mathrm{T}}\tilde{\mathcal{D}}^{1/2} - \mathcal{D}^{1/2}\mathcal{O}^{\mathrm{T}}\tilde{\mathcal{D}}^{-1/2})\Big] = \frac{1}{4}\Big[\tilde{\mathcal{D}}^{1/2}\mathcal{O}\mathcal{D}^{-1}\mathcal{O}^{\mathrm{T}}\tilde{\mathcal{D}}^{1/2} + \\ &+ \tilde{\mathcal{D}}^{1/2}\mathcal{O}\mathcal{O}^{\mathrm{T}}\tilde{\mathcal{D}}^{-1/2} + \tilde{\mathcal{D}}^{-1/2}\mathcal{O}\mathcal{O}^{\mathrm{T}}\tilde{\mathcal{D}}^{1/2} + \tilde{\mathcal{D}}^{-1/2}\mathcal{O}\mathcal{D}\mathcal{O}^{\mathrm{T}}\tilde{\mathcal{D}}^{-1/2} - \tilde{\mathcal{D}}^{1/2}\mathcal{O}\mathcal{D}^{-1}\mathcal{O}^{\mathrm{T}}\tilde{\mathcal{D}}^{1/2} + \\ &+ \tilde{\mathcal{D}}^{1/2}\mathcal{O}\mathcal{O}^{\mathrm{T}}\tilde{\mathcal{D}}^{-1/2} + \tilde{\mathcal{D}}\mathcal{O}\mathcal{O}^{\mathrm{T}}\tilde{\mathcal{D}}^{1/2} - \tilde{\mathcal{D}}^{-1/2}\mathcal{O}\mathcal{D}\mathcal{O}^{\mathrm{T}}\tilde{\mathcal{D}}^{-1/2}\Big] = \frac{1}{4}(4\mathbb{I}_{3N\times 3N}) = \mathbb{I}_{3N\times 3N} \end{aligned} \tag{S2}$$

and

$$\begin{aligned} &YX^T - XY^T = \frac{1}{4}\Big[\Big(\tilde{\mathcal{D}}^{1/2}\mathcal{O}\mathcal{D}^{-1/2} - \tilde{\mathcal{D}}^{-1/2}\mathcal{O}\mathcal{D}^{1/2}\Big)\Big(\mathcal{D}^{-1/2}\mathcal{O}^{\mathrm{T}}\tilde{\mathcal{D}}^{1/2} - \tilde{\mathcal{D}}^{-1/2}\mathcal{O}^{\mathrm{T}}\mathcal{D}^{1/2}\Big) + \\ &- (\tilde{\mathcal{D}}^{1/2}\mathcal{O}\mathcal{D}^{-1/2} + \tilde{\mathcal{D}}^{-1/2}\mathcal{O}\mathcal{D}^{1/2})(\mathcal{D}^{-1/2}\mathcal{O}^{\mathrm{T}}\tilde{\mathcal{D}}^{1/2} - \mathcal{D}^{1/2}\mathcal{O}^{\mathrm{T}}\tilde{\mathcal{D}}^{-1/2})\Big] = \frac{1}{4}\Big[\tilde{\mathcal{D}}^{1/2}\mathcal{O}\mathcal{D}^{-1}\mathcal{O}^{\mathrm{T}}\tilde{\mathcal{D}}^{1/2} + \\ &+ \tilde{\mathcal{D}}^{-1/2}\mathcal{O}\mathcal{O}^{\mathrm{T}}\tilde{\mathcal{D}}^{1/2} - \tilde{\mathcal{D}}^{-1/2}\mathcal{O}\mathcal{O}^{\mathrm{T}}\tilde{\mathcal{D}}^{1/2} - \tilde{\mathcal{D}}^{-1/2}\mathcal{O}\mathcal{D}\mathcal{O}^{\mathrm{T}}\tilde{\mathcal{D}}^{-1/2} - \tilde{\mathcal{D}}^{1/2}\mathcal{O}\mathcal{D}^{-1}\mathcal{O}^{\mathrm{T}}\tilde{\mathcal{D}}^{1/2} + \\ &+ \mathcal{D}^{1/2}\mathcal{O}\mathcal{O}^{\mathrm{T}}\tilde{\mathcal{D}}^{-1/2} - \mathcal{D}^{-1/2}\mathcal{O}\mathcal{O}^{\mathrm{T}}\tilde{\mathcal{D}}^{1/2} + \tilde{\mathcal{D}}^{-1/2}\mathcal{O}\mathcal{D}\mathcal{O}^{\mathrm{T}}\tilde{\mathcal{D}}^{-1/2}\Big] = 0. \end{aligned} \tag{S3}$$

II. C_{70} FULLERENE IN [8]-CPPA CYCLOPARAPHENYL RING MODEL

The supramolecular system of C_{70} fullerene surrounded by a cycloparaphenyl ring composed of 8 units consists of 166 atoms. DFTB+ simulations to calculate the ratios η_A were performed in cluster geometry (no periodic boundary conditions), with values for the net charge $Z_{tot} = 0$ and Fermi temperature $T = 0.001$ Ha.

III. SQ-MBD REPROJECTION SCHEME

The expectation value of the harmonic oscillator energy of atom A in the MBD ground state (for which $\hat{b}_k|\tilde{\mathbf{0}}\rangle = 0$) is given by

$$
\begin{aligned}
(U_{\mathrm{MBD}})_A &= \sum_{i=1}^{3} \hbar\omega_A \langle\tilde{\mathbf{0}}|\hat{a}^{\dagger}_{A_{x_i}}\hat{a}_{A_{x_i}}|\tilde{\mathbf{0}}\rangle = \sum_{i=1}^{3}\sum_{k,k'=1}^{3N} \hbar\omega_A (Y^T)_{A_{x_i}k}(Y^T)_{A_{x_i}k'}\langle\tilde{\mathbf{0}}|\hat{b}_k\hat{b}^{\dagger}_{k'}|\tilde{\mathbf{0}}\rangle = \\
&= \hbar\omega_A \sum_{i=1}^{3}\sum_{k=1}^{3N} Y^T_{A_{x_i}k}Y_{kA_{x_i}},
\end{aligned}
\tag{S4}
$$

while the expectation value of the interaction potential operator between two atomic QDOs is given by

$$
\begin{aligned}
(V_{\mathrm{MBD}})_{AB} &= \frac{1}{2}\sum_{i,j=1}^{3} \omega_A\omega_B(\mathcal{A}^{(0)}_A\mathcal{A}^{(0)}_B)^{1/2}(\mathbb{T}_{AB})_{ij}\langle\tilde{\mathbf{0}}|\hat{\boldsymbol{q}}_A\hat{\boldsymbol{q}}_B|\tilde{\mathbf{0}}\rangle = \frac{\hbar\omega_A^{-1/2}\omega_B^{-1/2}}{4}(\mathcal{A}^{(0)}_A\mathcal{A}^{(0)}_B)^{1/2} \\
&\times \sum_{i,j=1}^{3}(\mathbb{T}_{AB})_{ij}(\hat{a}_{A_{x_i}} + \hat{a}^{\dagger}_{A_{x_i}})(\hat{a}_{B_{x_j}} + \hat{a}^{\dagger}_{B_{x_j}}) = \\
&= \frac{\hbar\omega_A^{1/2}\omega_B^{1/2}}{4}(\mathcal{A}^{(0)}_A\mathcal{A}^{(0)}_B)^{1/2}\sum_{i,j=1}^{3}\sum_{k,k'=1}^{3N}(\mathbb{T}_{AB})_{ij}(X^T_{A_{x_i}k} - Y^T_{A_{x_i}k})(X^T_{B_{x_j}k} - Y^T_{B_{x_j}k})\langle\tilde{\mathbf{0}}|\hat{b}_k\hat{b}^{\dagger}_{k'}|\tilde{\mathbf{0}}\rangle = \\
&= \frac{\hbar(\omega_A\omega_B\mathcal{A}^{(0)}_A\mathcal{A}^{(0)}_B)^{1/2}}{4}\sum_{i,j=1}^{3}\sum_{k=1}^{3N} Q_{A_ik}Q^T_{kB_{x_j}}(\mathbb{T}_{AB})_{ij},
\end{aligned}
\tag{S5}
$$

where we have introduced the matrix $Q(X,Y) = X^T - Y^T$.

A. Comparison with reprojection scheme derived from ACFD-RPA description of many-body dispersion

The reprojection method for the MBD energy into single-fragment contributions is not unique. The MBD energy for a set of QDOs can be defined from the Adiabatic Connection Fluctuation Dissipation Theorem in Random Phase Approximation (ACFD-RPA) as [S2]

$$
\begin{aligned}
(E_{\mathrm{MBD}}) &= \frac{1}{2\pi}\int_0^{+\infty}\sum_{n=2}^{+\infty}\frac{(-1)^{n+1}}{n}\mathrm{Tr}[(\mathcal{A}^{(0)}(i\omega)\mathbb{T})^n]\mathrm{d}\omega = \frac{1}{2\pi}\int_0^{\infty}\mathrm{Tr}\left[\mathrm{Log}(\mathbb{I} + \mathcal{A}^{(0)}(i\omega)\mathbb{T})\right]\mathrm{d}\omega = \\
&= \sum_{A=1}^{N}\left[\frac{1}{2\pi}\int_0^{\infty}[\mathrm{Log}(\mathbb{I} + \mathcal{A}^{(0)}(i\omega)\mathbb{T})\mathrm{d}\omega\right]_{AA} = \sum_{A=1}^{N}(E^{\mathrm{ACFD}}_{\mathrm{MBD}})_A,
\end{aligned}
\tag{S6}
$$

where the condition $\mathrm{Tr}[\mathcal{A}^{(0)}\mathbb{T}] = 0$ has been used, it has been assumed that $\|\mathcal{A}^{(0)}\mathbb{T}\| < 1$ for some sub-multiplicative matrix norm (as, for instance, the Frobenius norm), and the logarithm of a matrix $B = \log(\mathrm{A})$ has been defined as usual such that $A = \exp(B) = \sum_{n=0}^{\infty} B^n/n!$.

A numerical consistency check between the reprojection of the MBD energy as defined in Eq.(S6) and the one derived from the SQ-MBD formalism, i.e. $(E_{\mathrm{MBD}}^{\mathrm{frag}})_A$, has been completed for the C70@8PPA supramolecular complex considered in the main text. The numerical integral over the frequency domain ω in (S6) has been evaluated using a trapezoid rule over the domain $\omega \in [0, 120]$ over finite intervals $\delta\omega = 0.002$ a.u. With this choice of integration parameters, the relative difference of the total MBD energy calculated from Eq.(S6) and the one calculated by the Hamiltonian MBD method is $\ll 10^{-7}$.

In Figure 1, the results are reported for the reprojection of the MBD energy defined from SQ-MBD and the relative difference with the ACFD-RPA method. The results clearly show that the two methods *are not equivalent*. The relative difference between the single-atom contributions is in the range of $-7.7\% \pm 4.1\%$ for each atom, with a more significant deviation in absolute value observed for the hydrogen atoms. We conjecture that the main difference between the two methods consists in the way the QDOs energy in the reference non-interacting ground state is distributed among the atoms. According to the reprojection procedure defined within the SQ-MBD method, the MBD energy of each 3D atomic QDO in the interacting system is calculated assuming its ground state energy in the non-interacting system as reference. On the other hand, the ACFD-RPA reprojection method integrates the energy differences between the non-interacting and interacting system without explicitly referring to each atom's non-interacting ground state energy. However, a detailed investigation of the inequivalence between the two methods in a more rigorous and systematic way is beyond the scope of the present work.

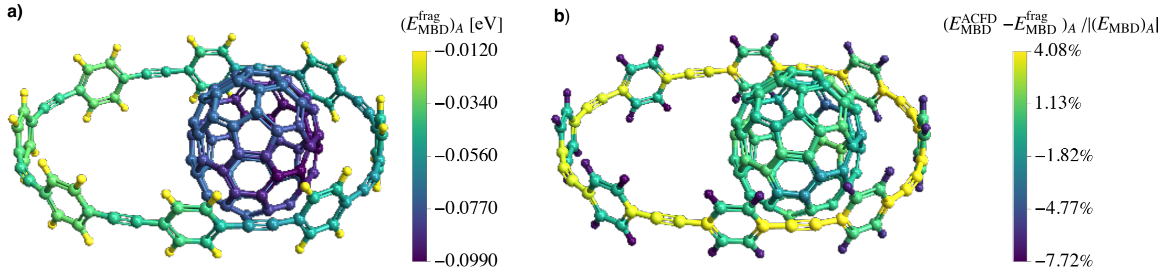

Supplementary Figure 1. **Comparison of projections method of the many-body dispersion (MBD) energy over atomic partition of C_{70} in [8]- CPPA cycloparaphenyl ring model**. In the panel a), the projection of the total MBD energy $(E^{\rm frag}_{\rm MBD})_A$ on the A-th atom within the SQ-MBD method is reported. In the panel b), the relative difference of the atomic contribution to MBD energy has been reported for two different projection methods: the second-quantized MBD projection energy $E^{\rm frag}_{\rm MBD}$ and the projection based on the connection fluctuation-dissipation theorem (ACFD) to many-body dispersion interactions relative to $(E^{\rm ACFD})_{\rm MBD}$. Source data are provided as a Source Data file.

IV. CRAMBIN MODEL

The structure of crambin was obtained from Protein Data Bank (PDB) file PDB-ID: 2FD7 and consists of 642 atoms. DFTB+ simulations to calculate the ratios η_A were performed in cluster geometry (no periodic boundary conditions), with values for the net charge $Z_{tot} = 0$ and Fermi temperature $T = 0.001$ Ha. To aid interpretation of the $V_{\rm MBD}$ matrices, we report here the distance matrices for geometrical barycenters of atomic positions for partitioning of crambin into residues (Figure S2) and into larger secondary structures (Figure S3). In the case of residue fragmentation, we can observe the correlation for the mutual distances between residue geometric centers of mass and their mutual MBD potential, with the interactions decaying as $\sim d^{-6.27}$. However, it is interesting to notice a deviation from such a power law for $d \lesssim 5\,\text{Å}$. This entails a two-fold explanation. For short length scales, the metric introduced to measure the distances between residues becomes less representative if the geometry of the partition, i.e., the reciprocal geometrical configuration of a pair of fragments, is poorly approximated by the geometrical centers of mass. On the other hand, such a deviation could be attributed to many-body effects among atomic QDOs belonging to the two fragments and with strong mutual correlations. For the secondary-structure par-

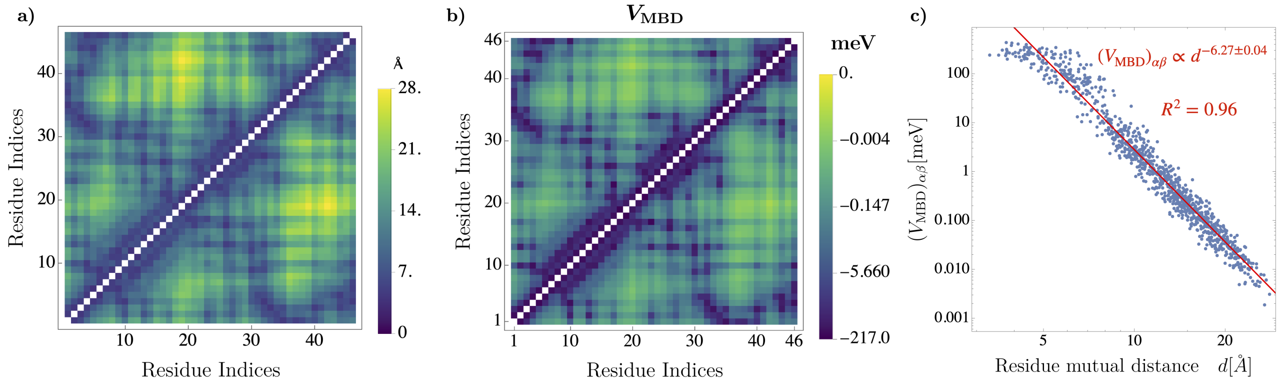

Supplementary Figure 2. **Geometric properties and mutual many-body dispersion (MBD) energy in crambin residue pairs**. In panel (a), the distances between geometrical centers of mass for all pairs of residues in crambin are shown. In panel b), the mutual interaction energies V_{MBD} for all pairs of residues are shown. In panel c), the correlation plot for the distances between geometrical centers of mass for all pairs α, β of residues and their mutual interaction energies is shown. Source data are provided as a Source Data file.

titioning, it is possible to recognize some common patterns between the matrices in Figures S3 (a) and b). However, in this case, the correlation plot reveals a much weaker correlation for the mutual MBD potential and the mutual distance between secondary structure motifs.

V. TRANSITION DIPOLES IN THE SQ-MBD FRAMEWORK

The contribution of atom A along the x_i Cartesian direction to the transition dipole from the MBD ground state $|\tilde{\mathbf{0}}\rangle \longrightarrow |\tilde{\mathbf{1}}_k\rangle$ associated with the single excitation of the k-th MBD mode is given in terms of X,Y matrices by

$$\mu_{\tilde{\mathbf{0}}\tilde{\mathbf{1}}_k;A_{x_i}} = \langle\tilde{\mathbf{1}}_k|\hat{\mu}_{A_{x_i}}|\tilde{\mathbf{0}}\rangle = \sqrt{\frac{\hbar\omega_A\mathcal{A}_A}{2}}(X-Y)^T_{A_{x_i}k}\,. \tag{S7}$$

VI. QUANTUM INFORMATION TOOLS FOR THE SQ-MBD MODEL OF QDOS

The MBD ground state as described in the SQ-MBD framework is a multimodal Gaussian bosonic state. One powerful method to investigate quantum information properties is based on the Williamson theorem [S3, S4]. Given an algebra of creation/annihilation operators, it

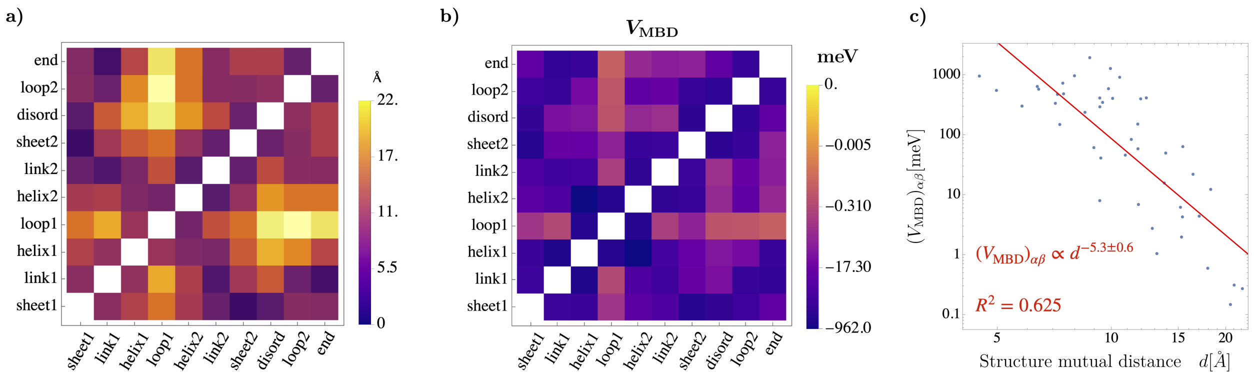

Supplementary Figure 3. **Geometric properties and mutual many-body dispersion (MBD) energy in crambin secondary-structure motifs.** In panel (a), the distances d between geometrical centers of mass for all pairs of secondary structures in crambin are shown. In panel b), the mutual interaction energies V_{MBD} for all pairs of secondary structures are shown. In panel c), the correlation plot for the distances between geometrical centers of mass for all pairs α, β of secondary structures and their mutual interaction energies is shown. Source data are provided as a Source Data file.

is possible to define the symplectic form

$$\Omega\hat{I} = \begin{pmatrix} [\hat{\boldsymbol{a}}, \hat{\boldsymbol{a}}] & [\hat{\boldsymbol{a}}, \hat{\boldsymbol{a}}^\dagger] \\ [\hat{\boldsymbol{a}}^\dagger, \hat{\boldsymbol{a}}] & [\hat{\boldsymbol{a}}^\dagger, \hat{\boldsymbol{a}}^\dagger] \end{pmatrix} = \begin{pmatrix} 0 & \boldsymbol{I} \\ -\boldsymbol{I} & 0 \end{pmatrix} \hat{I} \tag{S8}$$

and a group of symplectic transformations preserving the symplectic form such that

$$\Omega = \mathcal{S}^T \Omega \mathcal{S}\,. \tag{S9}$$

Within this framework, the Williamson theorem states that for any $2N_{\mathrm{frag}} \times 2N_{\mathrm{frag}}$ covariant matrix $\Sigma^{(\alpha)}$, there exists a symplectic matrix $\mathbf{S}$ such that

$$\Sigma^{(\alpha)} = \mathbf{S}\Sigma^{(\alpha)\oplus}\mathbf{S}^{\mathrm{T}} \qquad \Sigma^{(\alpha)\oplus} := \mathrm{diag}(\nu_1^\alpha, ..., \nu_{3N}^\alpha) \oplus \mathrm{diag}(\nu_1^\alpha, ..., \nu_{3N}^\alpha), \tag{S10}$$

where the N positive $\{\nu_k\}_{k=1,..3N}$ quantities are called symplectic eigenvalues. They can be easily calculated as the positive eigenspectrum of the matrix $i\Omega\Sigma^{(\alpha)}$. The von Neumann entropy associated to the reduced density matrix $\hat{\rho}_\alpha = \mathrm{Tr}_{\beta\neq\alpha}|\tilde{\mathbf{0}}\rangle\langle\tilde{\mathbf{0}}|$ can be calculated by

$$S(\hat{\rho}_\alpha) = \sum_{k=1}^{N_{\mathrm{frag}}} f(\nu_k^{(\alpha)}), \tag{S11}$$

where

$$f(x) = \left(\frac{x+1}{2}\right)\log\left(\frac{x+1}{2}\right) - \left(\frac{x-1}{2}\right)\log\left(\frac{x-1}{2}\right)\,. \tag{S12}$$

VII. CORRELATION BETWEEN POTENTIAL ENERGY AND MUTUAL INFORMATION FOR RESIDUE PAIRS

In order to establish how the mutual MBD interactions between fragment pairs influence their correlations, we report the correlation plot for the mutual information $(M.I.)_{\alpha\beta}$ of fragment pairs vs. $(V_{\mathrm{MBD}})_{\alpha\beta}$ in Figure S4. It can be noticed how such quantities are strongly correlated and carry the same information on the correlations among QDOs belonging to different fragments.

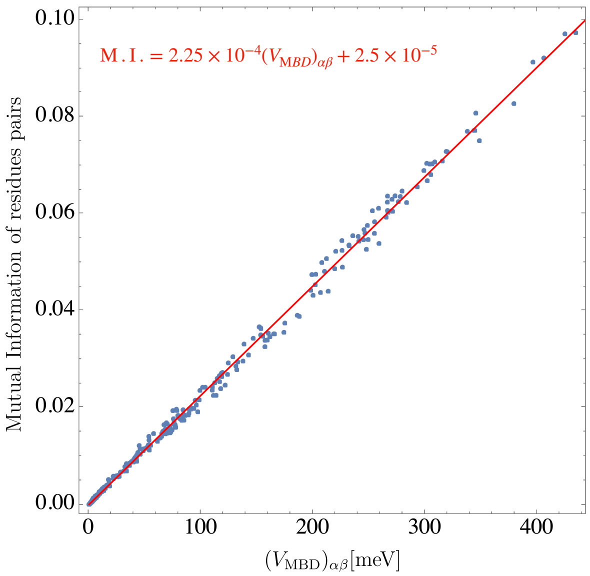

Supplementary Figure 4. **Correlation between mutual information and mutual interaction among different residues in crambin**. The figure shows the correlation plot for interfragment contribution among residues $(V_{\mathrm{MBD}})_{\alpha\beta}$ vs.the mutual MBD interactions M.I. between residue pairs for the crambin protein.Source data are provided as a Source Data file

VIII. SQ-MBD ANALYSIS OF THE S12L DATASET

The S12L is a dataset of systems stabilized by different kinds of supramolecular interactions and includes dispersion-dominated, π-stacked, hydrogen-bonded and cation–dipolar-bound structures supramolecular complexes contribute to binding energies [S5]. Such a dataset has been used to benchmark and validate a different method for estimating van

der Waals dispersion energies supramolecular complexes contribute to binding energies [S6]. and for showing how the analysis of MBD plasmon-like modes in supramolecular complexes contribute to binding energies [S7]. This section shows how the SQ-MBD method allows one to decompose information contained in the fully coupled MBD state of the whole system without the need for arbitrary projections into states of isolated fragments, as it is usually done in the first-quantized version of MBD [S7, S8].

A. Analysis of the MBD modes contribution to MBD interaction between fragments in dispersion-dominated and electrostatically-bound S12L structures

The SQ-MBD method allows one to easily evaluate the MBD mode contributions to the expectation value of the quadratic forms of the creation/annihilation operators for the atomic QDOs in the MBD ground state. This analysis reveals the contributions of single QDO excited states to the MBD ground state and the contribution of the MBD modes to collective variables.

Let us consider, for instance, the $(\hat{V}_{\rm MBD})_{\mathcal{AB}}$ operator describing the interaction between complex $\mathcal{A}$ and complex $\mathcal{B}$ for a given system in the S12L dataset in the equilibrium geometry of the complex. Thanks to the SQ-MBD expression of $(V_{\rm MBD})_{\mathcal{AB}} = \langle\tilde{\mathbf{0}}|(\hat{V}_{\rm MBD})_{\mathcal{AB}}|\tilde{\mathbf{0}}\rangle$ in Eq. (S5), it is possible to evaluate the contribution of each MBD mode to the inter-fragment MBD energy. We consider the same systems from the S12L database studied in [S7]: a "tweezer" complex dominated by non-polar dispersion interactions in Figure 5a), and an electrostatically bound cucurbituril complex in Figure 7a). In that case, the first quantization-based analysis was intended to identify the displacement component of each atomic QDO particle, giving the most important contributions to the MBD binding energy $E_{\rm bind,MBD} = (E_{\rm MBD})_{\mathcal{A}\cup\mathcal{B}} - [(E_{\rm MBD})_{\mathcal{A}} + (E_{\rm MBD})_{\mathcal{B}}]$, i.e., the difference between the total MBD energy of the complex and the MBD energy of the two fragments at an infinite distance. Such an analysis required the comparison of the given equilibrium configuration with another configuration (fragments at an infinite distance).

Here we propose an alternative observable, the two-fragment contribution to the MBD potential energy $(V_{\rm MBD})_{\mathcal{AB}}$, which is an inherent property of the MBD ground state in a given configuration. Thus, it does not require a comparison with other configurations. In particular, the SQ-MBD-based analysis we present investigates the contribution of single

MBD modes to the total inter-fragment MBD energy and the contribution of each pair of atomic QDOs to $(V_{\text{MBD}})_{\mathcal{AB}}$ for a given MBD mode.

1. The "tweezer" complex

In Figure S5 we have explored the contribution of each MBD mode to the interaction energy among fragments $(\hat{V}_{\text{MBD}})_{\mathcal{AB}}$ in the "tweezer" complex dominated by non-polar dispersion interactions. Given the relatively small size of the complex ($\sim 10^2$ atoms), atomic resolution has been adopted. For this complex, the total MBD binding energy is $E_{\text{bind,MBD}} \approx -0.818$ eV, while the inter-fragment interaction energy in the MBD ground state is $(V_{\text{MBD}})_{\mathcal{AB}} \approx -1.67$ eV. This difference arises because in SQ-MBD we compute the expectation value of the inter-fragment interaction energy evaluated on the fully coupled MBD state. The right panel in Figure S5 shows the almost equal presence of positive contributions of "anti-bonding" MBD modes and the negative contributions to the MBD interaction energy given by "bonding" MBD modes. It is relevant to note the distribution in the energy of these modes: the most negative one ($(V_{\text{MBD}})_{\mathcal{AB}} \approx -0.6$ eV) given by a low-frequency MBD eigenmode ($\hbar\tilde{\omega}_{\text{min}} \simeq 10.63$ eV) and the most positive one ($(V_{\text{MBD}})_{\mathcal{AB}} \approx +0.6$ eV) given by a high-frequency eigenmode ($\hbar\tilde{\omega}_{\text{max}} \simeq 17.99$ eV), while in the intermediate frequency range we observe a high density of modes giving an almost zero contribution to the interaction energy.

The normalized MBD eigenvectors for both the strongest "bonding" and "anti- bonding" MBD modes are reported in panels on the right side of Figure 6. The MBD eigenvector associated with the "bonding" MBD eigenmode $\tilde{\omega}_{\text{min}}$ defines a "solenoidal"-like vector field over the atoms in the complex. In contrast, the MBD eigenvector associated with $\tilde{\omega}_{\text{max}}$ seems to exhibit an "irrotational"-like vector field. The SQ-MBD formalism allows one to easily estimate and visualize the contribution of each pair of atomic QDOs to $(V_{\text{MBD}})_{\mathcal{AB}}$ for a given MBD mode. In particular, it is possible to notice that in the eigenmode $\tilde{\omega}_{\text{min}}$, the interaction energy between the two complexes is minimized, as demonstrated by the predominance of blue regions in Figure 6a), but this is compensated by the increased MBD interaction energy among the atoms in the eigenmode $\tilde{\omega}_{\text{max}}$, as demonstrated by the predominance of green and yellow regions in Figure 6c).

2. The electrostatically bound cucurbituril complex

An analogous MBD modal analysis has been performed on the cucurbituril system, where electrostatic interactions dominate the bound energy (see Figure S7). In this case, the bonding energy between the cucurbituril ring and the smaller complex at its center ($E_{\text{bind,MBD}} \approx -1.890$ eV) and the MBD interaction energy ($(V_{\text{MBD}})_{\mathcal{AB}} \approx -1.865$ eV) are almost equal. Both "bonding" and "antibonding" MBD modes have been found, with the most prominent "bonding" mode ($(V_{\text{MBD}})_{\mathcal{AB}} \sim -0.4$ eV) in the low-frequency region at $\hbar\tilde{\omega}_{\text{min}} \sim 11$ eV and the most prominent "antibonding" mode ($(V_{\text{MBD}})_{\mathcal{AB}} \sim +0.6$ eV) in the high-frequency region at $\hbar\tilde{\omega}_{\text{max}} \sim 21$ eV. However, "antibonding" MBD modes have also been found in this case in a middle-low frequency range $\hbar\tilde{\omega} \approx 12.5$ eV associated with QDOs excitations among the terminus of the complex inside the ring, and "bonding" MBD modes in the middle-high frequency range $\hbar\tilde{\omega} \approx 17.5$ eV associated with the collective excitation between each of the phenyl groups and half of the cucurbituril ring. The SQ-MBD-based analysis of the most prominent "bonding" MBD mode in Figure S8a) shows a more homogeneous pattern of the matrix with respect to the previous "tweezer" system. This can be interpreted as a consequence of the high quasi-cylindrical symmetry of the system: the coherence between the atomic QDOs in the cucurbituril ring and the QDOs in the smaller complex does not prevent coherences between the atomic QDOs in the fragments. Such an analysis is complemented by the geometrical visualization provided in Figure S8b), where the normalized MBD eigenvector is reported on the real geometry, also showing in this case a "quasi-solenoidal" vector field. On the contrary, the SQ-MBD-based analysis of the most prominent "antibonding" MBD mode in Figure S8c) reveals a strong coordination pattern among atoms in the larger complex that does not coordinate with the QDOs in the smaller complex at the center of the ring. Figure 8d) reveals the geometry of the "irrotational"-like vector field of the normalized displacements associated with the "antibonding" modes.

a) b)

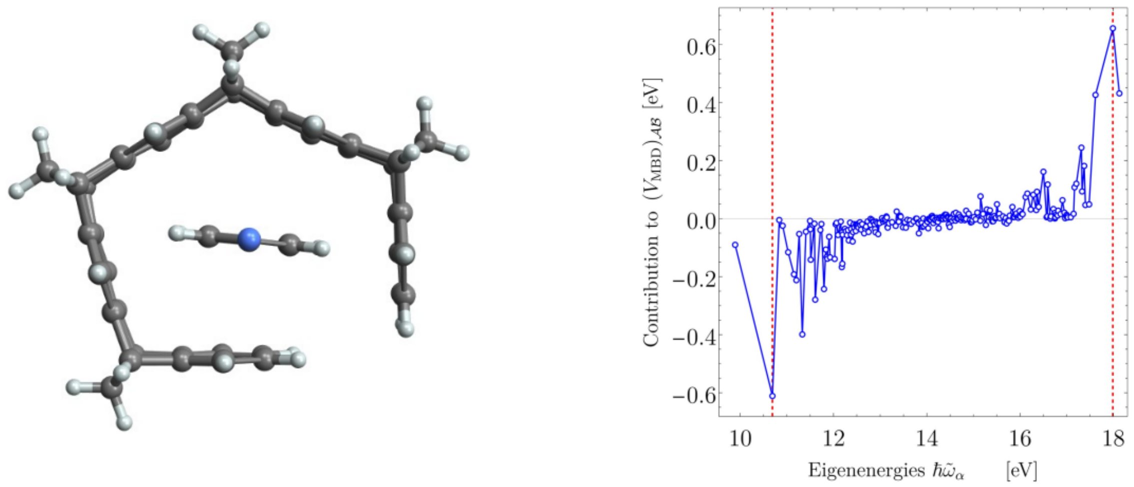

Supplementary Figure 5. **Inter-fragment MBD potential energy** $(V_{\mathrm{MBD}})_{\mathcal{AB}}$ **in the "tweezer" complex with 1,4-dicyanobenzene, dominated by non-polar dispersion interactions**. In panel (a), the chemical structure of the complex is shown, and in panel b) the MBD eigenmode energies $\hbar\tilde{\omega}_{\mathrm{MBD}}$ are plotted vs. the contribution of each mode to the inter-fragment MBD potential energy $(V_{\mathrm{MBD}})_{\mathcal{AB}}$ among two arbitrary fragment $\mathcal{A}, \mathcal{B}$. The red dashed lines highlight the MBD eigenenergies giving the most negative (left line) and most positive (right line) contributions to $(V_{\mathrm{MBD}})_{\mathcal{AB}}$.Source data are provided as a Source Data file

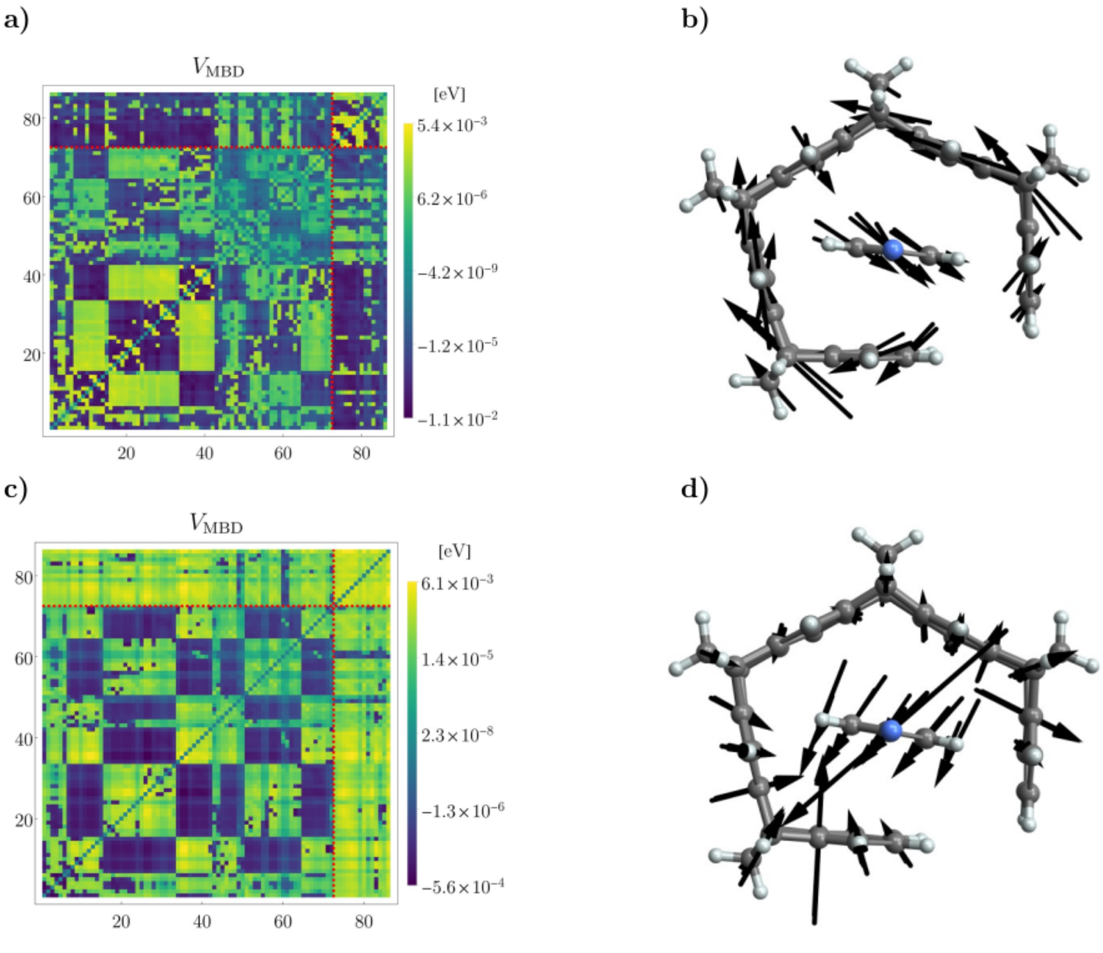

Supplementary Figure 6. **Analysis of many-body dispersion (MBD) modes contributions to the interfragment MBD energy $(V_{\mathrm{MBD}})_{\mathcal{AB}}$ in the "tweezer" complex with 1,4-dicyanobenzene, dominated by non-polar dispersion interactions.** In the upper panels, the V_{MBD} matrix at atomic resolution in (a) and the components of the normalized MBD eigenvector in b) are reported for the MBD mode giving the most negative contribution to the inter-fragment MBD potential energy. In the lower panels, the V_{MBD} matrix at atomic resolution in c) and the components of the normalized MBD eigenvector in d) are reported for the MBD mode giving the most positive contribution to the inter-fragment MBD potential energy. The dashed (perpendicular) red lines in the V_{MBD} matrices separate the sets of atoms belonging to the two unlinked fragments in the complex. Source data are provided as a Source Data file

a) b)

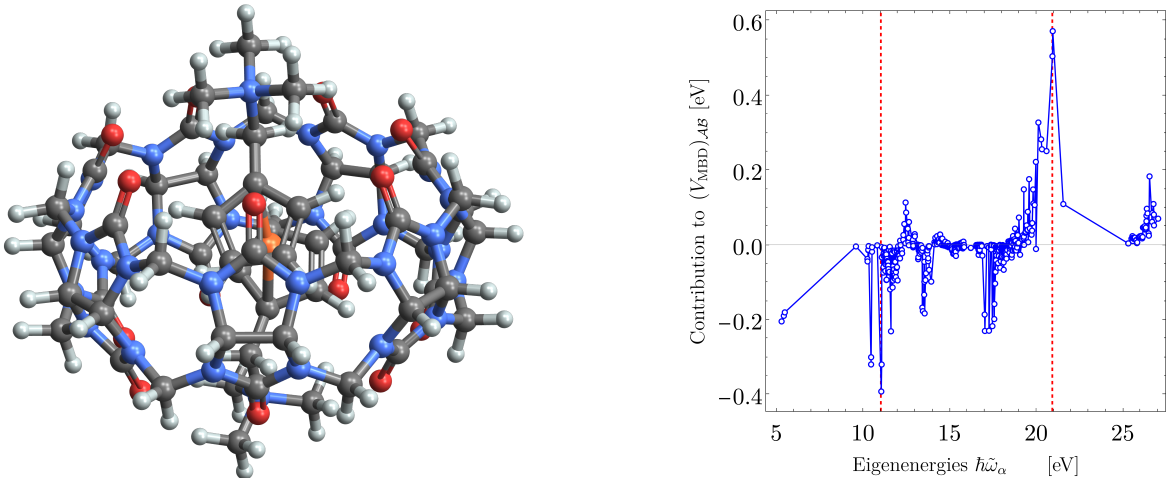

Supplementary Figure 7. **Inter-fragment MBD potential energy $(V_{\mathrm{MBD}})_{\mathcal{AB}}$ in the electrostatically bound cucurbituril complex**. In panel (a) the chemical structure of the complex [S5] is shown, with the iron ion Fe^{2+} displayed in orange at the center. In panel b) the MBD eigenmode energies $\hbar\tilde{\omega}_{\mathrm{MBD}}$ are plotted vs. the contribution of each mode to the inter-fragment MBD potential energy $(V_{\mathrm{MBD}})_{\mathcal{AB}}$ among two arbitrary fragment $\mathcal{A}, \mathcal{B}$. The red dashed lines correspond to the MBD eigenenergies giving the most negative (left line) and most positive (right line) contributions to inter-fragment MBD potential energy.Source data are provided as a Source Data file

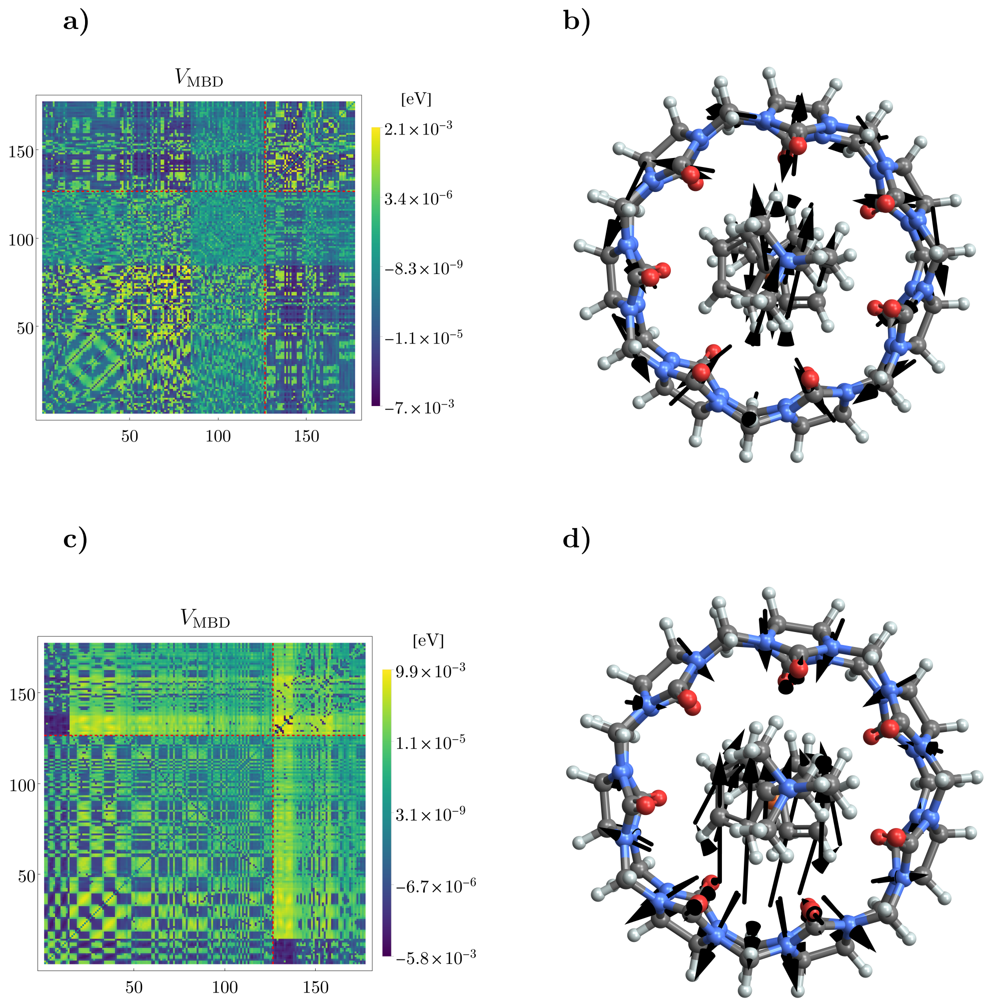

Supplementary Figure 8. **Analysis of many-body dispersion (MBD) modes contributions to the interfragment MBD energy** $(V_{\mathrm{MBD}})_{\mathcal{AB}}$ **in the electrostatically bound cucurbituril complex.** In the upper panels, the V_{MBD} matrix at atomic resolution in a) and the components of the normalized MBD eigenvector in b) are reported for the MBD mode giving the most negative contribution to the inter-fragment MBD potential energy. In the lower panels, the V_{MBD} matrix at atomic resolution in a) and the components of the normalized MBD eigenvector in b) are reported for the MBD mode giving the most positive contribution to the inter-fragment MBD potential energy. The dashed (perpendicular) red lines in the V_{MBD} matrices separate the sets of atoms belonging to the two unlinked fragments in the complex.Source data are provided as a Source Data file

[S1] Ripka, S., Blaizot, J. & Ripka, G. *Quantum Theory of Finite Systems* (MIT Press, Cambridge, 1986). URL `https://books.google.com/books?id=s\_xlQgAACAAJ`.

[S2] Tkatchenko, A., Ambrosetti, A. & DiStasio, R. A. Interatomic methods for the dispersion energy derived from the adiabatic connection fluctuation-dissipation theorem. *J. of Chem. Phys.* **138**, 074106 (2013).

[S3] Weedbrook, C. *et al.* Gaussian quantum information. *Rev. Mod. Phys.* **84**, 621 (2012).

[S4] Adesso, G., Ragy, S. & Lee, A. R. Continuous variable quantum information: Gaussian states and beyond. *Open Syst. Inf. Dyn.* **21**, 1440001 (2014).

[S5] Grimme, S. Supramolecular binding thermodynamics by dispersion-corrected density functional theory. *Chem.Eur.J.* **18**, 9955–9964 (2012).

[S6] Grimme, S., Hansen, A., Brandenburg, J. G. & Bannwarth, C. Dispersion-corrected mean-field electronic structure methods. *Chem. Rev.* **116**, 5105–5154 (2016).

[S7] Hermann, J., Alfe, D. & Tkatchenko, A. Nanoscale π–π stacked molecules are bound by collective charge fluctuations. *Nat. Commun.* **8**, 14052 (2017).

[S8] Stöhr, M., Van Voorhis, T. & Tkatchenko, A. Theory and practice of modeling van der waals interactions in electronic-structure calculations. *Chem. Soc. Rev.* **48**, 4118–4154 (2019).
